# The current status and influence factors of research ability among community nurses: A sequential qualitative–quantitative study

**DOI:** 10.1515/med-2025-1314

**Published:** 2025-10-29

**Authors:** Yi Qin, Li Yan, Ying Zhang, Jordan Tovera Salvador, Linlin Liu

**Affiliations:** Faculty of Nursing, Guangxi University of Chinese Medicine, Nanning, Guangxi, 530200, China; Wuzhou Gongren Hospital, Wuzhou, Guangxi, 528400, China; Department of Nursing Education, College of Nursing, Imam Abdulrahman Bin Faisal University, Dammam, Saudi Arabia; Faculty of Nursing, Guangxi University of Chinese Medicine, No.13 Wuhe Avenue, Qingxiu District, Nanning, Guangxi, 530200, China

**Keywords:** nurses, nursing research, community health nursing, public health

## Abstract

**Background and aim:**

The study used a sequential qualitative–quantitative research design to develop a survey tool that explores the current situation and influencing factors of community nurses’ research ability.

**Methods:**

The qualitative research developed the Chinese Community Nurses Research Ability Tool (CCN-RAT) through face-to-face interviews. The quantitative research tests the current situation and influencing factors of research ability among community nurses. One-way ANOVA and Multiple Linear Regression were used to analyze the results.

**Results:**

The Cronbach’s *α* was found to be 0.993, and the Subject-Content Validity Index was 0.99 using CCN-RAT. Most of the community nurses had a low to moderate level of research ability. Age (95%CI: 1.812–9.533, *P* = 0.004), educational attainment (95%CI: 4.667–11.660, *P* < 0.001), working experience year (95%CI: 0.274–1.0410, *P =* 0.001), number of participants in research projects (95%CI: 0.239–11.130, *P* = 0.041), number of published articles in Chinese core journal or SCI (95%CI: 19.354–36.969, *P* < 0.001), and number of participants in research training (95%CI: 18.289–28.218, *P* < 0.001) were significant influencing factors on research ability among community nurses.

**Conclusion:**

CCN-RAT could help nurses, educators, students, and other populations to better understand the current situation and influencing factors of research ability, which could provide targeted research training to improve research ability for public health staff.

## 1 Introduction

Positive aging has led to most of China’s elderly moving into residential communities. In China, individuals and their families are now at the center of the healthcare system. Research in the field of nursing could help find answers to many of the issues plaguing the field of community health nursing. As a result, research aptitude influences nursing research directly [1]. While nursing research in the Chinese community still has a gap compared with community health nursing in developed countries, community nurses have low awareness of nursing research and low research ability in Shanghai City [2]. Professionalism in researching and the ability to conduct research are two necessities for community nurses. Expertise in research includes the ability to recognize research issues, gather and analyze data, apply to an Institutional Review Board (IRB), write and publish an article in either Chinese or English, and distribute one’s work both nationally and internationally. Writing a study proposal, carrying it out, getting it approved by an IRB, publishing the results in journals, and using them in practice are all part of the research process. Because Chinese community nurses’ research skills are not up to the minimum acceptable level for conducting research at either the national or international level, there is a great need for community nurses to engage in research. It is important to focus on and investigate the elements that influence the development of research skills among Chinese community nurses.

Most community nurses are frontline workers who have amassed extensive work experience and recognize the value of clinical data research. However, there is a shortage of research literacy among community nurses. Community nurses in China have yet to be assessed for and trained in their research abilities. The assessment tool of research ability among community nurses is lacking in China and in other countries. Pinar & Ozlem developed a tool, Scientific Research Competency Scale (SRCS), in Turkey, which was aimed to develop a valid and reliable scale in order to identify the scientific research competencies of nursing professionals at undergraduate and graduate levels [3], while SRCS was not suitable to test the research ability of Chinese community nurses according to different research emphasis, different target population, and different research requirements. Most studies regarding developing instruments of nurses’ research capacity were published after 2009, focusing on nurses and nursing students. Additional studies were necessary to enhance and assess the internal consistency and validity of the nurse’s research capacity instrument. The development process should incorporate qualitative interviews with the target population, specifically focusing on gauging reliability, comprehensiveness, and understandability of content validity within these instruments, encouraging the researchers to explore the development of more reliable and feasible instruments for different nursing populations based on a unified concept of nursing research competence, aimed to build a “gold standard” instrument [4]. In view of the suggestions in the literature above, Chinese Community Nurses’ Research Ability Tool (CCN-RAT) should be completed and developed to further understand the current situation of research ability among community nurses. In this way, the elements that affect community nurses’ ability to conduct research will be identified and presented, allowing the appropriate administrative body to focus on them, take the necessary measures, and craft policies that will improve community nurses’ research prowess in China. Researchers in this study hoped that by involving a diverse sample of participants, they would be able to generate fresh insights on how to evaluate the research skills of Chinese community health nurses. The purpose of this study is to develop a more reliable and validity new tool of research ability among community nurses and to explore the research ability of Chinese community nurses via the new tool developed in this study.

## 2 Research paradigm

**Figure 1:**
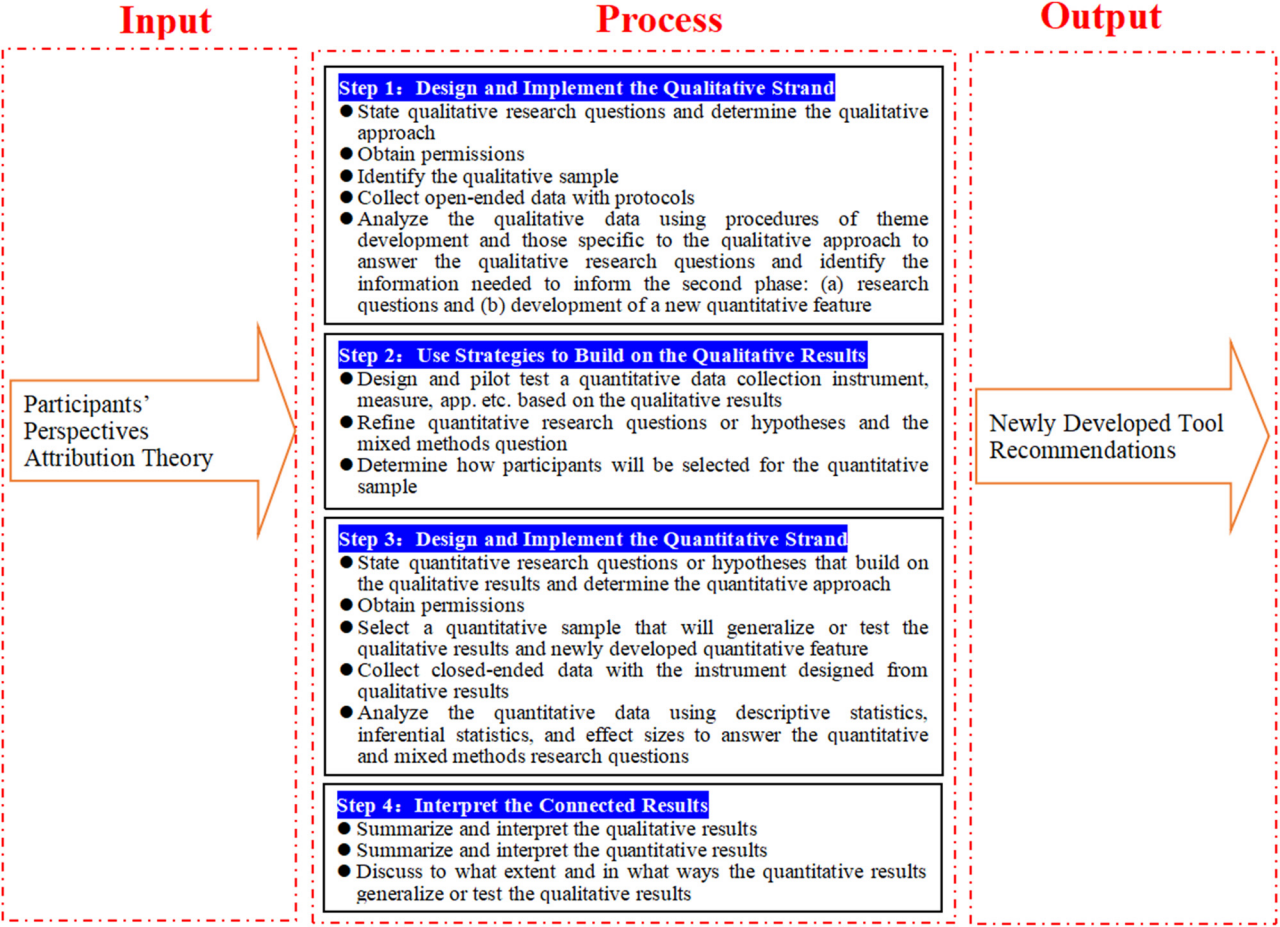
Research paradigm.

The research paradigm is presented via a Systems Theory Model (STM). STM in this study consists of three parts, including input, process, and output (Figure 1). The input part includes participants’ perspectives and attribution theory. The process part is the flowchart of the basic procedures in implementing a sequential qualitative–quantitative study design. It consists of four steps: Step 1 designs and implements the qualitative strand; Step 2 uses strategies to build on the qualitative results; Step 3 designs and implements the quantitative strand; and Step 4 interprets the connected results. The output part concerns newly developed tools and recommendations. Input, process, and output are interactive and interdependent parts. A change in one part can affect the other parts or the whole research. For example, participants’ perspectives may affect the development of new tools and recommendations.

## 3 Methods

### 3.1 Study design

In this work, qualitative and quantitative data were collected and analyzed sequentially using a sequential qualitative–quantitative study design [5,6]. The sequential qualitative–quantitative study design is a research approach where qualitative data collection and analysis are conducted first, followed by quantitative data collection and analysis. This design ensures that quantitative research is well-informed by real-world insights, improving both relevance and rigor. First, qualitative data were gathered from 15 Chinese community health nurses to better comprehend their perception of the researchers’ abilities to conduct the study. The newly produced tool formed in phase 1 was then applied to 6 PhD nursing specialists in order to assess validity, and to 35 community nurses in order to test dependability. Third, the highly valid and reliable tool developed for this study was used to get insight into the existing state of affairs and the factors impacting research aptitude among 178 community nurses.

### 3.2 Participants and sampling

Phase 1 – Qualitative Design. For the sample size, as reported by Morse [7] and Berband [8], at least 6 participants should be chosen for phenomenological studies. Bertaux labeled 15 as the smallest acceptable sample size in qualitative research [9]. Therefore, 15 registered community nurses who met the following criteria were recruited using purposive sampling for the qualitative study: (1) they were Chinese registered nurses employed by a public or private community health nursing facility; (2) they had at least three years of relevant work experience; and (3) they were willing to cooperate with the investigation. This study excluded community nurses due to their mental illness and their refusal to take part in the research.

Phase 2 – Tool Development. Purposive sampling was used to choose 35 community nurses and 6 nursing experts to test the validity and reliability of the survey instruments used in the tool development process. Doctoral experts with 10 years or more of working experience have richer research experience to interview research questions and can provide more scientific suggestions for modification when developing scales. Therefore, there were six eligible nurses who participated with the following inclusion criteria: (1) at least 10 years of experience in the field, (2) a strong background in nursing, (3) a doctorate in nursing, and (4) a willingness to take part in the study. Thirty-five community nurses were eligible for inclusion if they met the following criteria: (1) they were part of the official nursing staff at a Community Health Service Center (CHSC); (2) they had at least 1 year of relevant work experience; and (3) they were willing to cooperate with the inquiry.

Phase 3 – Quantitative Design. Nanning, Guangxi, China, residents were chosen at random for the quantitative study. There were 50 Community Health Service Centers in Nanning. One hundred and four community nurses were included in the study according to the following formula for determining the sample size:

\[n=\frac{{S}^{2}}{{(\varepsilon /z)}^{2}}.]\]

*S* represents the standard deviation, and according to a previous study, the standard deviation of research ability among clinical nurses was 23.53 [10]. *Z* represents a 95% confidence interval, and was equal to 1.96. *ε* represents the permitted error and is equal to 5. *n* represents the sample size in this study and is calculated as follows:

\[n=\frac{{23.53}^{2}}{{(5/1.96)}^{2}}=85.07\approx 86.]\]

As a result, 86 community nurses served as the study’s primary sample size. The sample size, 86 community nurses, should be increased by 20% to account for the attrition rate of 20% on the survey. Since 86 + 18 = 104, the total number of participants in this study was 104. This indicates that a total of 104 community nurses were included in the sample. In the Nanning City in the Guangxi Zhuang Autonomous Region, China, at least 104 community nurses were randomly chosen. Purposive sampling was used to establish the sample size. Eight towns in two of Nanning’s seven districts provided the participants, all of whom fulfilled the study’s inclusion requirements. The following were included in the study: The CHSC’s official nursing staff averaged a year of experience in the field per employee. In this research, we excluded nurses with less than a year of experience in the field and nurses diagnosed with a mental illness. Twenty-two to twenty-three nurses were chosen by purposive sampling from each of eight communities in two different districts. Therefore, in an effort to lower the impact of bias in this study, 178 participants were chosen via purposive sampling.

### 3.3 Research instruments

Data collection consisted of interviews, field notes, and audio recordings with a semi-structured format. These methods were selected to facilitate an in-depth investigation into the impact of the participants’ professional backgrounds on their capacity for research.

The instrument for testing the validity of survey tools included face validity and the content validity index (CVI). Face validity criteria of the survey tool were concerned with the appropriateness of grammar, clarity, and unambiguity of items, correct spelling of words, correct structuring the sentences, appropriateness of font size, and the structure of the instrument in terms of construction and well-thought-out format; the nursing experts provided the recommendations based on the face validity criteria. The CVI was used to invite nursing experts to rate the scores of the relevant items in the research. The rating score ranging from 1 to 4: 1 indicated not relevant, 2 = somewhat relevant, 3 = relevant, and 4 = very relevant. The instrument for testing the reliability of the survey tool was developed based on the recommendations provided by six nursing experts, named the Chinese community nurses’ research ability tool (CCN-RAT). It included 6 domains, which referred to literature review ability, identify problem’s ability, research design ability, research practice ability, data analysis ability, and manuscript writing ability, with 35 items.

The instrument applied in the quantitative study was developed based on the results of phase 1 and phase 2, named CCN-RAT, which is the same instrument applied in testing the reliability of survey tools in this study.

### 3.4 Data collection

The researchers submitted the relevant documents for the application for approval for collecting data from the Institutional Review Board of the Philippine Women’s University (Approval no. ERB2022_0024). Prior to commencing the study, researchers contacted and visited with participants, emailed them detailed information about the study, and explained all the steps to them. The informed permission was freely provided by participants after they had fully grasped all aspects of the investigation.

In this investigation, researchers conducted two interviews. The first interviews were face-to-face from July 13, 2022 to July 14, 2022, with the researcher asking questions from an open-ended questionnaire, detail by detail, for 15 participants. The record was implemented by the researcher (QY), including sound recordings by phone, observation, field notes, and documents/artifacts. The whole process took around 15–30 min per survey, and all record data were checked by another researcher (LLL). Based on the results of the first-round interview, the questionnaire (CCN-RAT) was designed and created. The researcher kept meticulous records of the interviews, including phone recordings, field notes, and any relevant documents or artifacts they came across. The questionnaire (CCN-RAT) was developed based on the findings from the initial interview. Thirty-five community nurses were employed to assess CCN-RAT’s reliability, and six international nursing experts were interviewed to determine the validity of the tool prior to the start of the follow-up interview from July 18, 2022, to August 3, 2022. The second interview was conducted to assess the current state and influential factors of research ability among community nurses in Nanning, Guangxi, China, using the CCN-RAT developed based on the findings of the first interview from August 5, 2022, to August 12, 2022. The CCN-RAT was distributed to the nurses, who were given clarification by the researcher if they had any questions.

### 3.5 Data analysis

To analyze the data, the Colaizzi technique was used to collect and interpret data by the researcher. Transcripts and analysis results were shared with participants once the researcher had completed each of Colaizzi’s seven procedures for data analysis. In doing so, the students were able to correct grammatical and typographical issues, add more information to their posts, and elaborate on their initial words and phrases. Consolidated Criteria for Reporting Qualitative Data (COREQ) were used to ensure the accuracy of the findings.

The second phase of the study was the quantitative phase, starting with the self-development survey procedure. The results of the phenomenological-based approach generated theme domains, which were used as constructs as the six headings for the large-scale sections within the survey tool. In the instrument, the categories were clusters of themes and codes produced from the qualitative data sets that were utilized as the individual survey items. The most exemplified qualitative data categories were chosen as survey items to keep the survey as concise as possible, similarly surveying the most essential constructs in the qualitative findings. Therefore, all emergent contextual categories were represented as survey response items. The self-developed tool was subjected to validity (face and CVI) and reliability test (internal consistency and split-half test). Data analysis was performed using SPSS Version 27.0.

The third phase was analyzed using descriptive statistics (percentage, mean, frequency distribution, etc.). Spreadsheets were utilized to collate all pertinent data to determine the total average of the newly developed survey tool.

### 3.6 Ethical consideration

Ethical permission for this sequential qualitativequantitative study was granted by the Philippine Women’s University (PWU) (Approval no. ERB2022_0024). The study followed the Declaration of Helsinki. The approval from the PWU was required for a study conducted in China because ethical and institutional review is based on the affiliation of the researcher, not solely the location of the research. When a student or faculty member from PWU conducts any research, whether domestically or internationally, they must adhere to the ethical principles and oversight procedures mandated by their home institution. PWU’s Institutional Review Board (IRB) is responsible for ensuring that its researchers uphold international ethical standards, which prioritize participant safety, informed consent, and data integrity, regardless of where the study takes place. This study is not part of any institutional cooperation or local exemption.

## 4 Results

### 4.1 Themes of CCN-RAT emerged in qualitative research (phenomenological strategy)

Fifteen community nurses who fulfilled the inclusion criteria were surveyed; all participants were female and employed in the field of community health nursing. Work experience ranged from 3 to 23 years, education from lower than a bachelor’s degree was common, and professional titles included nurse practitioner, supervising nurse, and associate professor of nursing.

**Figure 2:**
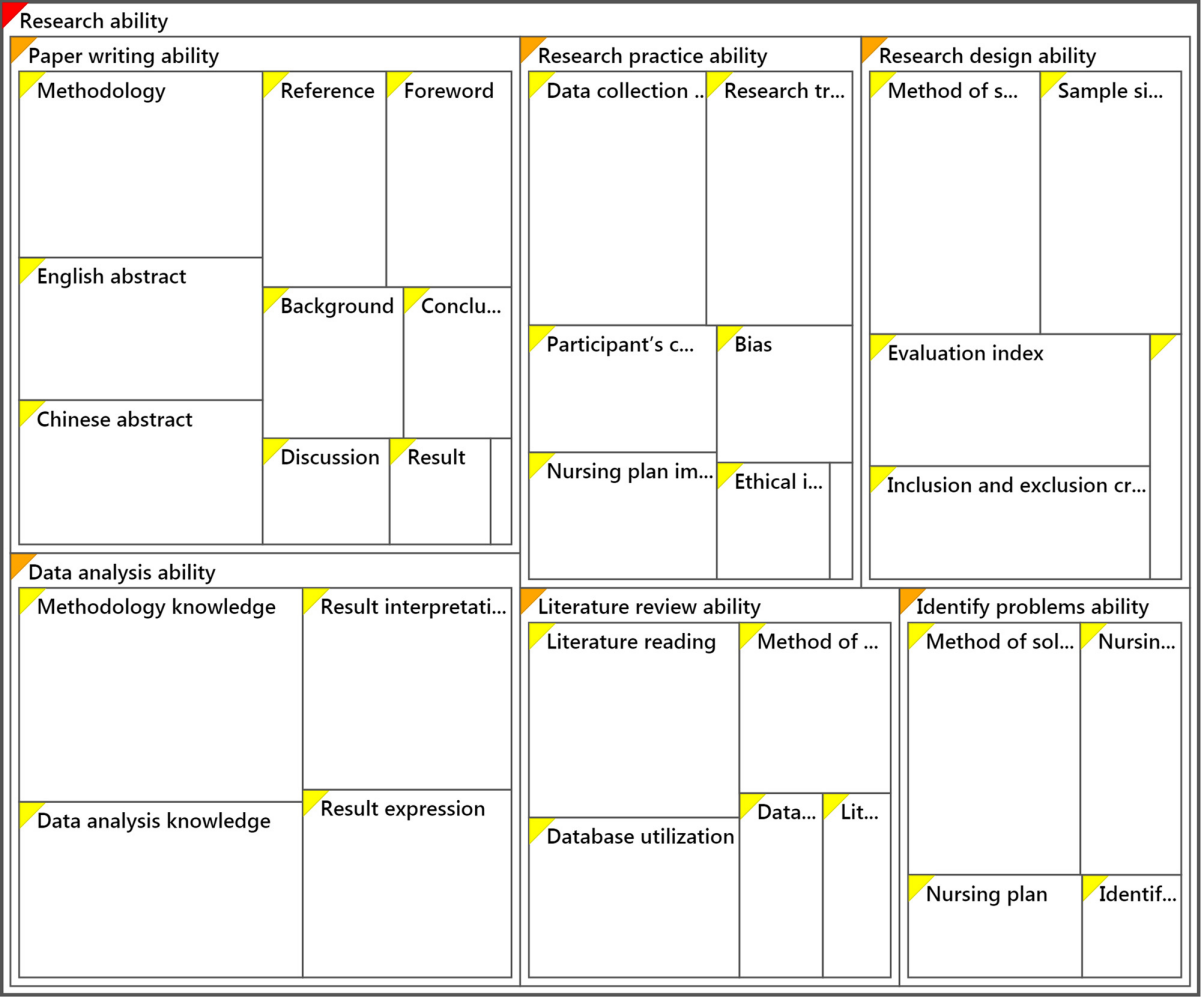
Hierarchy chart of research ability.

**Figure 3:**
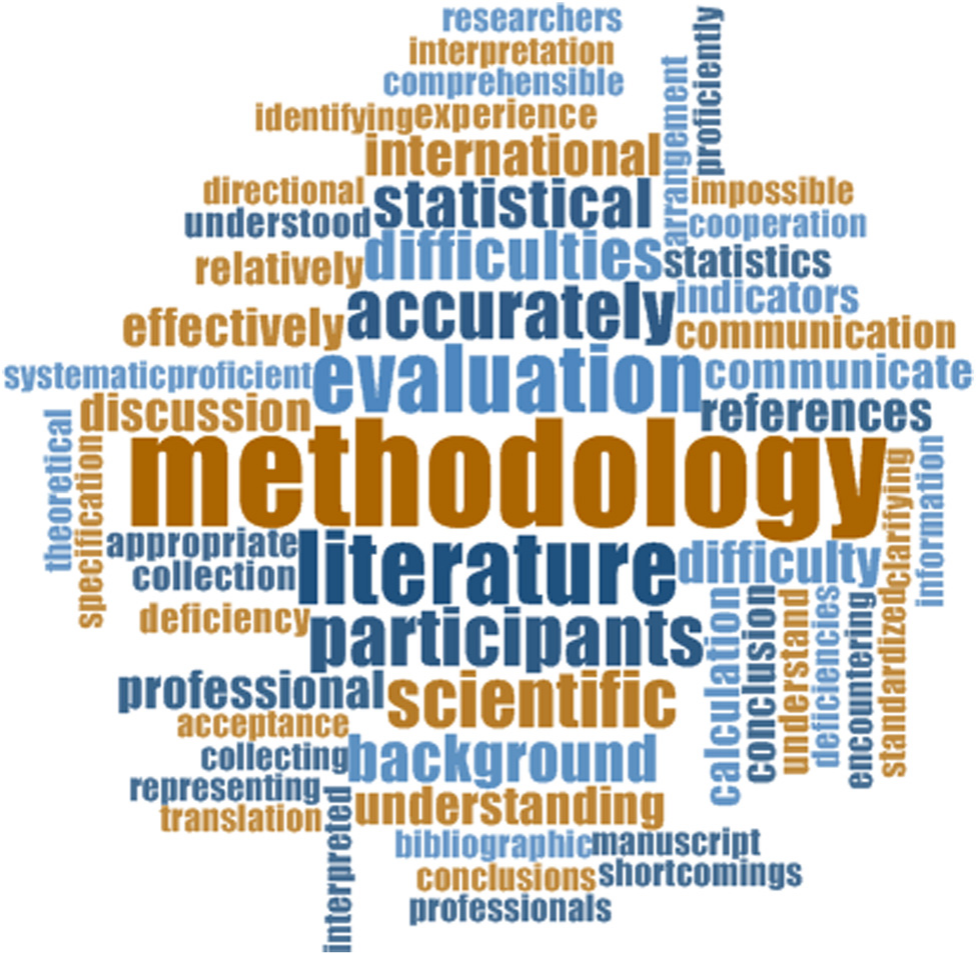
Word frequency of the difficulties of research.

Colaizzi’s technique identified the process of analysis of a qualitative study, including data analysis, major themes, theme clusters, formulated meaning, and significant statements. The researcher summarized six major themes from the narratives of 15 participants (Table 1, Figure 2), which included one literature review ability. The excerpts from participants’ statements were as follows: P1: “I have difficulty in domestic and international databases, searching relevant data, because I seldom search relevant data”, P6: “ I can’t read relevant literature in English due to poor English, which may produce difference understanding”; (2) identify problems with ability. The excerpts from participants’ statements were as follows: P2: “It is difficult to propose a plan to solve nursing problems, and can’t find out and understand the relevant theory to guide and solve nursing problems”, P9: “We need to be guided to identify nursing problems. Some guidance from a leader or other teachers may be required”; (3) research design ability. The excerpts from participants’ statements were as follows: P1: “I don’t know how to calculate sample size by relevant formula? and I don’t know how to identify which suitable method to select sample size”, P5: “It is difficult for me to calculate the sample size and I don’t know what should be evaluation indicators”; (4) research practice ability. The excerpts from participants’ statements were as follows: P1: “We don’t have a chance to do nursing research, so it is difficult to make a nursing research plan, because I haven’t practiced in this field”, P8: “There will be some deficiencies in the ability to deal with things, and there is no such a good solution in the program. It is difficult to provide a nursing plan to solve problems due to poor working experience”; (5) data analysis ability. The excerpts from participants’ statements were as follows: P1: “It is difficult to write methodology part as heavy work and limited time to learn it”, P3: “I can’t use graphs to describe results, and don’t know how to use methodology to analyze and describe results”; and (6) manuscript writing ability. The excerpts from participants’ statements were as follows: P4: “Manuscript writing is also now the part we are missing, because the staff in community health centers seldom publish articles, I think it is difficult to have time to write an article”, P12: “It is difficult for me to write background, abstract in English version, methodology, result, summary, reference in normative style due to poor research experience.” The top five frequency words expressed by participants were methodology, literature, evaluation, participants, and scientific (Figure 3), which indicates that the difficulties of research for community nurses are about methodology, literature review, evaluation of research, participants’ communication, and scientific research.

**Table 1:** Matrix of the tool development

Thematic construct	Cluster of themes	No. of items	Survey concept	Survey construct	Survey items (based on the narratives of the participants)
Literature review	Database knowledge	5	**Literature review ability**	Database knowledge	Ability to familiarize with domestic and international databases
Database usage and reading literature	Utilization of the database			Database utilization	Ability to review relevant community nursing literature by using a database
	Ability to read literature			Literature reading	Ability to read community nursing literature in English
	Ability to search literature by using a correct searching strategy			Method of searching the literature	Ability to accurately search community nursing literature with keywords
	Ability to analyze literature			Literature analysis	Ability to accurately analyze community nursing-related literature
Identify problems	Ability to identify problems	4	**Ability to identify problems**	Identify problems	Ability to identify problems in community nursing work
Find and solve problems	Know the way of solving nursing problems			Method of solving the problem	Ability to use a suitable method to solve problems
	Ability to make a nursing plan as a solution			Nursing plan	Ability to make a nursing plan to solve problems encountered in community nursing work
	Ability to choose a correct nursing theory to guide and solve nursing problems			Nursing theory	Ability to find the relevant theory to guide and solve problems
Research design	Calculation of sample size	5	**Research design ability**	Sample size calculation	Ability to calculate sample size accurately using a formula or SPSS
Sample size, inclusion and exclusion criteria, evaluation index, and result evaluation	Using the correct method to calculate the sample size			Method of sample size calculation	Ability to select the suitable sample size calculation method
	Ability to make inclusion and exclusion criteria for participants			Inclusion and exclusion criteria	Ability to define the scope of inclusion and exclusion of participants
	Ability to make an evaluation index			Evaluation index	Ability to define the evaluation index of research
	Ability to know how to evaluate the research results			Evaluating results	Ability to clear evaluation criteria for research results
Research practice	Ability to implement a nursing research plan	7	**Research practice ability**	Nursing plan implementation	Ability to develop a community nursing research plan
Nursing plan, ethical issue, bias, data collection and analysis, research training, communication, and research leader	Ability to consider ethical issues			Ethical issue	Ability to consider ethical issues at any time during research
	Ability to reduce bias			Bias	Ability to effectively reduce bias in the process of research
	Accurately collect and analyze data			Data collection and analysis	Ability to collect and analyze relevant data accurately
	Ability to provide research training			Research training	Ability to provide professional research training for research team members
	Ability to communicate with participants			Participant’s communication	Ability to effectively communicate and cooperate with research subjects
	Ability to be a research leader			Research leader	Ability to guide the research team member to conduct research
Data analysis	Data analysis knowledge	4	**Data analysis ability**	Data analysis knowledge	Ability to present results in tables and charts
Methodology use	Methodology knowledge			Methodology knowledge	Ability to analyze data by using correct statistical methods
	Ability to interpret the results			Result interpretation	Ability to accurately interpret statistical analysis results
	Ability to express results			Result expression	Ability to accurately describe statistical results
Paper writing	Ability to write a foreword	10	**Paper writing ability**	Foreword	Ability to write the foreword of a paper
Formatting of articles	Ability to write an abstract in Chinese			Chinese abstract	Ability to write an abstract in Chinese
	Ability to write an English abstract			English abstract	Ability to write an abstract in English
	Ability to write keywords			Keywords	Ability to write keywords in papers
	Ability to write a background			Background	Ability to write the background of the paper
	Ability to write methodology			Methodology	Ability to write the methodological part of the paper
	Ability to write results			Result	Ability to write the results of the paper
	Ability to write a discussion section			Discussion	Ability to write the discussion section of the paper
	Ability to write a conclusion section			Conclusion	Ability to write the discussion section of the paper
	Ability to write a reference section			Reference	Ability to write the reference section of the paper

Bold data represent the themes come from qualitative results.

### 4.2 Higher values of validity and reliability of CCN-RAT

**Figure 4:**
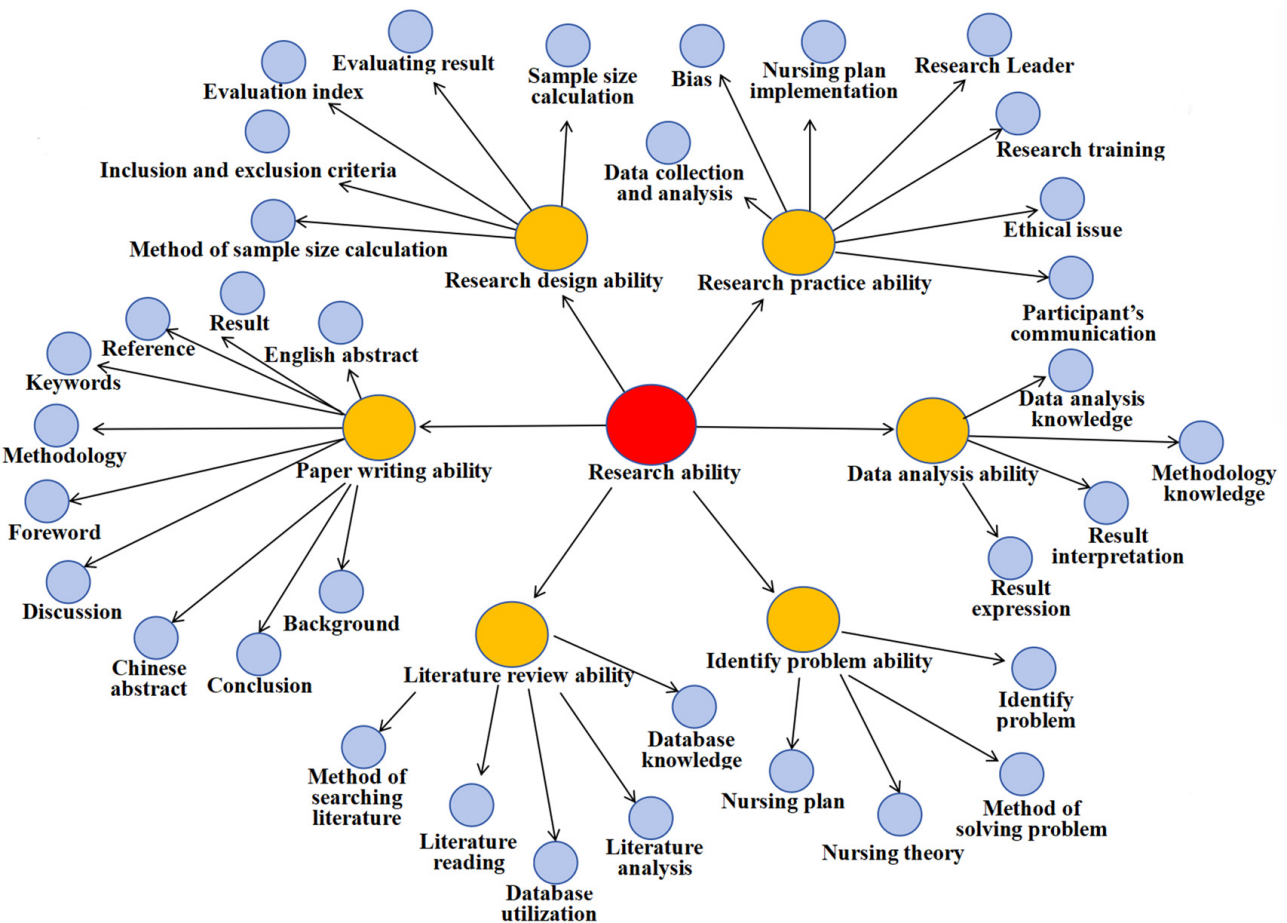
Project map of research ability.

Each item of the survey questionnaire was produced based on the results of face-to-face interviews during qualitative research were formed and considered as individual survey items. The results of phase 1 qualitative research based on the phenomenological approach manifested in six themes that were utilized as the main domains of the survey questionnaire. To keep the survey as short as feasible, only the most representative categories of qualitative data were selected as survey items during face-to-face interviews, capturing the most crucial aspects in qualitative findings. As a result of this qualitative research, we were able to identify six categories and 35 items as potential survey response options. Table 1 displays the correlation between the survey question and the qualitative results (Figure 4).

For the purpose of creating a new survey tool, a panel of PhD nurses was enlisted. The six panels’ combined experience covered adult nursing, nursing curriculum and teaching and administration, nursing science and nursing for urinary diseases, nursing for the elderly, and nursing management and community health. Their members hailed from China, Thailand, and Indonesia. There was a wide variety of employment experience, from 13 to 28 years.

Face validity is determined by a review of the survey items. Face validity in this study was analyzed by the suggestions and comments provided by six panels. The criteria for providing comments were derived from survey criteria of Oluwatayo [11]: (1) appropriateness of grammar, (2) clarity and unambiguity of items, (3) correct spelling of words, (4) correct structuring the sentences, (5) appropriateness of font size, and (6) structure of the instrument in terms of construction and well-thought-out format (Table 2).

**Table 2:** Comments from six panels

Appropriateness of grammar	Panel’s qualitative comments	Panel reviewers
Clarity and unambiguity of items	(1) No. 1 is not clear. What is familiar to domestic and international that researchers want? (2) No. 13 is not clear (3) Please check the details in Item No.10, because the sample size can be calculated by the formula or some program such as GPower or other programs. In the case of SPSS, it cannot be used for the calculation of sample size (4) Item No. 20 is not fit with your samples, they have a minimum of 3 years working and are not the principal investigator or head of research project, should change it to “Ability to attend professional research training”. (5) Item No. 22, same comments as Item No. 20, they are not the head of the research project	1, 5, 6
Correct spelling of words	(1) No. 26: It should be “an article”	1
Correct structuring the sentences	(1) Item No. 18 should separate into two items	6
Appropriateness of font size	(1) Should increase font size to 12 point	2
Structure if the instrument in terms of construction and well-thought out format	It would be better if there was the process of entering the research area, such as getting to know the community leaders, key information, etc.It will be better that all items should not be covered or duplicated, such as No. 18Please check and change the sequence of each item followed by the ability in the research process, utilization or dissemination, after that make a sequence by the ability to support the other community nurse or research team members.No. 4 item should appear before item No. 3No. 8 item should appearbefore item No. 7	3, 4, 5, 6

### 4.3 CVI for subject-content validity index (S-CVI)

The I-CVI and average S-CVI were used to determine content validity in this study. Each survey item was assigned a relevant score between 1 and 4 (1 = not relevant, 2 = somewhat relevant, 3 = relevant, and 4 = highly relevant) by six panels of nursing experts. All experts agreed or disagreed on whether 35 items fit the study’s principal concept and issue. After compiling, assessing, and summarizing the feedback from the PhD in nursing panel, we made some changes to the survey instrument, including removing one item and adding another. In the end, 35 items were selected to comprise the final iteration of the self-improvement survey instrument, with items 19 and 21 having an I-CVI value of 0.83 and the other items having an I-CVI value of 1. In this analysis, the self-improvement survey instrument had an S-CVI of 0.99.

After receiving positive feedback from all six validators on the survey’s content validity, the researcher immediately launched a pilot study to gauge the instrument’s dependability. In order to express reliability, an online survey was conducted with 35 participants between 05 and 09 August 2022, and the results were analyzed for internal consistency.

The data of reliability tests implemented by a pilot study were collected and analyzed using Statistical Package for Social Sciences (SPSS) 27.0 version. The Cronbach’s *α* and split-half test in SPSS were utilized to test the statistical measurement of reliability, and internal consistency was the content to express reliability in this study. The Cronbach’s *α* in this study was 0.993. The results of the split-half test in this study were 0.986 (Part 1) and 0.990 (Part 2); part 1 items were 1, 2, 3, 4, 5, 6, 7, 8, 9, 10, 11, 12, 13, 14, 15, 16, 17, and 18 and part 2 items were 19, 20, 21, 22, 23, 24, 25, 26, 27, 28, 29, 30, 31, 32, 33, 34, and 35. According to the interpretations of internal consistency from George and Mallery [12], the internal consistency of a total of 35 respondents in this study was excellent, which meant the 35 items of the self-developed survey tool were more reliable to assess the research ability among community nurses.

The survey tool entitled Chinese Community Nurses Research Ability Tool (CCN-RAT) includes two parts: (1) demographic information and (2) research ability assessment. The demographic profile consisted of age, gender, marital status, educational attainment, professional post, year of working experience in community, monthly income, job title, number of presiding research project, number of participants in the research project, number of published articles, number of published articles in a Chinese core journal or SCI, and number of participants in research training. The research ability assessment part consisted of six domains of research ability, which were produced in the narrative of community nurses in phase 1, including literature review ability, identifying problems’ ability, research design ability, research practice ability, data analysis ability, and manuscript writing ability.

### 4.4 Application of CCN-RAT in exploring the *status quo* and influence factors of research ability

On the basis of relevant factors of research ability, the demographic profiling were considered as the final entries. The detailed demographic profiles of 178 participants are listed in Table 3.

**Table 3:** Demographic profile of the participants; *n* = 178

Demographic profile description	Contents	*N* (%)	Mean ± SD
Age	20–29	51 (28.7%)	
	30–39	63 (35.3%)	
	40–49	51 (28.7%)	
	50–59	13 (7.3%)	
Gender	Men	3 (1.7%)	
	Women	175 (98.3)	
Marital status	Single	32 (18.0%)	
	Married	132 (74.1%)	
	Divorced/annulled	13 (7.3%)	
	Separated	1 (0.6%)	
Educational attainment	Diploma	19 (10.7%)	
	Associate degree	75 (42.1%)	
	Bachelor’s degree	83 (46.6%)	
	Master’s degree	1 (0.6%)	
	Doctoral degree	0	
Professional title	Registered nurse	39 (21.9%)	
	Nurse practitioner	61 (34.3%)	
	Nurse-in-charge	69 (38.8%)	
	Associate professor of nursing	9 (5.0%)	
	Professor of nursing	0	
Years of working experience in the community	1–5 years	32 (18.0%)	14.49 ± 8.827
	6–10 years	46 (25.8%)	
	11–20 years	55 (30.9%)	
	More than 20 years	45 (25.3%)	
Monthly income	<1,000 Yuan	1 (0.6%)	
	1,001–5,000 Yuan	128 (71.9%)	
	5,001–10,000 Yuan	44 (24.7%)	
	>10,000 Yuan	5 (2.8%)	
Job title	Community nurse	149 (83.7%)	
	Community head nurse	12 (6.7%)	
	Director of Nursing Department	17 (9.6%)	
Number of presiding in research projects	0	154 (86.5%)	0.21 ± 0.572
	1	10 (5.6%)	
	2	14 (7.9%)	
	3	0	
	4	0	
Number of participants in research projects	0	146 (82.1%)	0.27 ± 0.634
	1	18 (10.1%)	
	2	12 (6.7%)	
	3	2 (1.1%)	
	4	0	
Number of published articles	0	141 (79.2%)	0.29 ± 0.623
	1	23 (12.9%)	
	2	13 (7.3%)	
	3	1 (0.6%)	
Number of published articles in Chinese core journals or SCI journals	0	172 (96.6%)	0.04 ± 0.222
	1	5 (2.8%)	
	2	1 (0.6%)	
	3	0	
Number of participants in research training in the past one year	0	71 (39.9%)	0.61 ± 0.501
	1	106 (59.6%)	
	2	1 (0.5%)	
	3	0	

Note. SD = standard deviation; SCI = science citation index; *N* = Number.

The survey tool included 35 items with 5 options based on Likert’s five-grade scoring method [13], including highly unacceptable, unacceptable, moderately acceptable, acceptable, and highly acceptable, resulting in a 1–5 score. Therefore, the total score of the survey tool ranged from 35 to 175, with a score of 35–81 indicating a low level of research ability, 82–128 moderate level of research ability, and 129–175 high level of research ability. Based on the results of the level of research ability, 59 (33.2) community nurses had low-level research ability, 112 (62.9) community nurses had moderate-level research ability, and only 7 (3.9) community nurses presented a high level of research ability. The detail information is shown in Table 4.

**Table 4:** Total scores and the level of research ability among community nurses; *N* = 178

Total score of research ability	Level of research ability	*N* (%)	Mean ± SD
35–81	Low	59 (33.2%)	92.60 ± 25.521
82–128	Moderate	112 (62.9%)	
129–175	High	7 (3.9%)	

Note. SD = standard deviation; *N* = number.

One-way ANOVA was applied to understand the influencing factors of research ability among community nurses. Age, marital status, educational attainment, professional title, year of working experience, monthly income, job title, number of presiding research projects, number of participants in research projects, number of published articles, number of published articles in a Chinese core journal or SCI, and number of participants in research training in the past 1 year had statistically significant influence on the research ability among community nurses (*P* < 0.05) (Table 5).

**Table 5:** One-way ANOVA on research ability among community nurses; *N* = 178

Demographic profile description	Contents	Mean ± SD	*t*/*F* value	*P*
Age	20–29	64.86 ± 19.703	61.203	**<0.001**
	30–39	98.38 ± 19.166		
	40–49	107.41 ± 15.988		
	50–59	115.23 ± 9.688		
Gender	Men	86.67 ± 47.816	3.587	0.848
	Women	92.70 ± 25.212		
Marital status	Single	67.38 ± 21.749	17.779	**<0.001**
	Married	96.92 ± 23.516		
	Divorced/annulled	108.38 ± 9.683		
	Separated	124.00 ± 0.000		
Educational attainment	Diploma	86.26 ± 30.371	5.983	**0.001**
	Associate degree	85.23 ± 27.094		
	Bachelor’s degree	100.33 ± 20.235		
	Master’s degree	124.00 ± 0.000		
	Doctoral degree	0		
Professional title	Registered nurse	69.51 ± 26.813	31.215	**<0.001**
	Nurse practitioner	88.13 ± 23.033		
	Nurse-in-charge	106.54 ± 14.008		
	Associate professor of nursing	116.00 ± 17.507		
	Professor of nursing	0		
Years of working experience in the community	1–5 years	62.38 ± 20.41	46.497	**<0.001**
	6–10 years	85.00 ± 22.651		
	11–20 years	101.51 ± 21.003		
	More than 20 years	110.96 ± 9.537		
Monthly income	<1,000 Yuan	105.00 ± 0.00	8.306	**<0.001**
	1,001–5,000 Yuan	86.97 ± 26.359		
	5,001–10,000 Yuan	106.89 ± 15.548		
	>10,000 Yuan	108.40 ± 23.480		
Job title	Community nurse	90.52 ± 26.649	4.147	**0.017**
	Community head nurse	95.42 ± 18.672		
	Director of Nursing Department	108.82 ± 8.376		
Number of presiding research projects	0	90.62 ± 25.813	5.233	**0.006**
	1	94.40 ± 24.690		
	2	113.07 ± 10.171		
	3	0		
	4	0		
Number of participants in research projects	0	89.83 ± 25.884	0.174	**<0.001**
	1	101.11 ± 24.588		
	2	108.67 ± 7.703		
	3	121.50 ± 3.536		
	4	0		
Number of published articles	0	87.74 ± 25.554	9.487	**<0.001**
	1	111.39 ± 17.908		
	2	109.62 ± 7.077		
	3	124.00 ± 0.000		
Number of published articles in Chinese core journals or SCI journals	0	91.19 ± 24.748	8.440	**<0.001**
	1	133.40 ± 10.139		
	2	131.00 ± 0.000		
	3	0		
Number of participants in research training in the past 1 year	0	68.70 ± 23.133	124.328	**<0.001**
	1	108.30 ± 9.836		
	2	124.00 ± 0.000		
	3	0		

Note. SD = standard deviation; SCI = science citation index.

Bold data represents the data is less than 0.05, it has a significant value.

Statistically significant independent variables of the results of one-way ANOVA were selected as independent variables. The total score of research ability was chosen as the dependent variable. Multiple linear regression analysis was applied and calculated to evaluate the influencing factors of research ability among community nurses.

The results showed that age, educational attainment, year of working experience, number of participants in research projects, number of published articles in Chinese core journals or SCI, and number of participants in research training in the past 1 year were statistically significant and the influencing factors on research ability among community nurses (*P* < 0.05) (Table 6).

**Table 6:** Multiple linear regression analysis on research ability among community nurses; *N* = 178

Demographic profile description	*B*	Standard error	Standard coefficient	*t*	*P*	95%CI
Age	5.673	1.955	0.205	2.901	**0.004**	1.812∼9.533
Marital status	−0.545	2.411	−0.011	−0.226	0.821	−5.305∼4.215
Educational attainment	8.163	1.771	0.217	4.609	**<0.001**	4.667∼11.660
Professional title	0.958	1.773	0.032	0.540	0.590	−2.543∼4.459
Years of working experience in the community	0.657	0.194	0.227	3.386	**0.001**	0.274∼1.041
Monthly income	2.803	2.047	0.058	1.369	0.173	−1.238∼6.843
Job title	−0.799	1.899	−0.019	−0.421	0.674	−4.548∼2.949
Number of presiding research projects	1.867	2.785	−0.042	−0.670	0.504	−7.366∼3.633
Number of participants in research projects	5.684	2.758	0.141	2.061	**0.041**	0.239∼11.130
Number of published articles	−3.648	2.814	−0.089	−1.297	0.197	−9.204∼1.907
Number of published articles in Chinese core journals or SCI journals	28.161	4.461	0.245	6.313	**<0.001**	19.354∼36.969
Number of participants in research training in the past one year	23.253	2.514	0.457	9.248	**<0.001**	18.289∼28.218

Note. CI = confidence interval; SCI = science citation index.

Bold data represents the data is less than 0.05, it has a significant value.

## 5 Discussion

Upon examining the narratives of the participants based from the data analysis of Colaizzi [14], six major themes arise that flashes back the perspectives of the participants towards the difficulties of conducting a research that the community nurses should possess in improving research ability in nursing in the future, namely: (1) literature review ability, (2) identify problems ability, (3) research design ability, (4) research practice ability, (5) data analysis ability, and (6) manuscript writing ability. The results of phase 1 qualitative research based on a phenomenological approach appeared in six themes, which were utilized as the main domains of the survey questionnaire. The survey questionnaire was both tested in phase 2 and used in phase 3; therefore, qualitative research results provided reliable data and a basis for the development of CCN-RAT and the exploration of the current situation and influencing factors of community nurses’ research ability.

Research ability among nurses has received extensive attention worldwide. Nursing has gradually become a scientific discipline. The nursing discipline requires its own knowledge system and best evidence-based practice, which is based on excellent research ability [15]. In addition, evidence-based practices have spread worldwide. Nurses nowadays are the major healthcare professionals to provide nursing care and have the responsibility to provide high-quality care based on the best evidence [16]. Many countries and organizations have tried to enhance research ability in the nursing discipline [17]. While there are insufficient policies to support the improvement of research ability in the nursing discipline, which could be an intervention to improve research ability, more studies have paid more attention to how to improve nurses’ research ability [18].

Most of Nanning City’s community nurses had a moderate or low level of research experience, according to the findings of this study. Among community nurses in Nanning City, factors such as age, level of education, years of experience, number of research projects participated in, number of articles published in Chinese core journals or SCI, and recent participation in research training were found to have a significant impact on researchers’ abilities. The research ability among community nurses needs to be improved.

The global nursing profession increasingly recognizes that high-quality, evidence-based care depends upon strong research capacity, a need highlighted by the finding that most community nurses in Nanning City possess only a moderate or low level of research experience. This local data underscores the gap between the discipline’s scientific aspirations and the on-the-ground reality. Factors such as education level, research training, and publication history were identified as significant influencers on research ability in Nanning, confirming that targeted interventions are crucial. Therefore, improving research capacity among these nurses is not just a local concern but a vital step for advancing evidence-based practice within the broader nursing discipline.

Many community nurses in Nanning City, Guangxi, China, were found to have a light to moderate research background, according to the study’s findings. Nanning is the capital of the Guangxi Zhuang Autonomous Region, and compared to other cities in the region, it offers superior conditions for carrying out a research project in terms of funding, research training opportunities, and institutional backing, among other factors. It seems to be the reason that community nurses in other Guangxi Zhuang Autonomous Region will have less research prowess.

Many Chinese institutions and organizations have called for increased participation by nurses in high-quality nursing research as a condition for accreditation or promotion. One problem was that the research base was not being used to inform policy or practice. There is a disconnect between theory and practice in clinical nursing, as evidence-based practice is notoriously challenging [18]. Landeen performed research on the impact of an HRCoP with the intention of fostering the growth of nursing research capability [18]. The findings demonstrated that the primary question served to boost participants’ self-assurance and skill in planning and carrying out a research study. The research participation rate among nurses was shockingly low, at around 43%. According to reports, the driving forces behind nurses’ success in carrying out studies and publishing their findings were their enthusiasm, dedication, and mental fortitude [18]. Gullick and West addressed that a community of 25 advanced practice nurses who had more than 7 years had a positive influence on their members’ research capacity and productivity [19]. It was proposed that the faculty generally holds a stronger research ability, including research skill, knowledge, and experience, which was foundational to successful research projects within nursing. Clinicians could recognize the most urgent requirements, but it did not mean that the clinicians held the ability to explore innovative solutions or to effectively utilize the best evidence-based practice [20]. As a result, a collaborative strategy between clinical nurse leaders and academic institutions has been created to help nurses improve their research skills. Equally helpful in building skills and self-assurance for conducting original research was involvement in community practice [18].

As was previously mentioned, the lack of resources (financial, human, and otherwise) at community health service centers in Nanning City makes it difficult for community nurses to conduct research, especially those with less experience and a bachelor’s or associate degree. The course of nursing research was not included in the curriculum provided for a college degree or vocational degree. Furthermore, community health service centers rarely offer the research course or appropriate research training to improve community nurses’ research abilities, such as their knowledge, skills, and experience in conducting independent research and publishing articles. As the average age of community nurses increased, so did their opportunities for engaging in research, as well as their capacity for recognizing nursing issues; also, the higher a nurse’s professional rank, the greater the demand for research. As a result, it is possible that senior community nurses have greater research abilities than their younger counterparts.

The year of work experience is explained in the same way as age. More years in the workforce mean more opportunities to perform studies. A senior nurse with years of expertise is often sought out to take part in research and contribute to a report. Especially for the manuscript published in a Chinese core journal or Science Citation Index (SCI) publication, this may increase the head nurse’s awareness and expertise in conducting research. Articles accepted for publication in a Chinese core journal, or a SCI journal, are held to a higher standard and must adhere to stricter guidelines in every respect. The “total number of published articles” is a simple measure of productivity but does not account for quality or impact. A high count could include publications in low-quality or predatory journals with minimal scientific scrutiny. In contrast, “articles in core/SCI journals” act as a proxy for quality and impact. These journals employ a rigorous peer-review process, ensuring that the research meets higher methodological and significance standards. Publishing in them is more difficult and competitive. Therefore, a positive association with core/SCI publications suggests that it is not merely the act of publishing, but the ability to produce work that meets a high scholarly benchmark, that is linked to stronger research ability. This distinction separates routine activity from demonstrable scholarly excellence. Therefore, characteristics such as age, years of experience in the field, the size of the research team, and the number of articles published in Chinese core journals or SCI journals may all affect a community nurse’s capacity for original research.

Community nurses’ research skills are strongly influenced by their level of education. Many community nurses have associate degrees or diplomas in nursing, whereas fewer have bachelor’s degrees. However, undergraduates pursuing a bachelor’s degree are constantly urged to take on leadership roles in research projects. All of this might give master’s level nursing students, in particular, greater opportunities to apply their research skills, while also helping bachelor’s level students understand more about research. Master’s degree candidates in nursing are tasked with conducting original research and publishing the results in peer-reviewed journals. Master’s degree students have access to a comprehensive curriculum covering nursing research, including Nursing Research, Medical Methodology, and Nursing English. As a corollary, it stands to reason that the more the educational level of community nurses, the greater their capacity for research. Short addressed that the practitioners received a research course in a bachelor’s degree but may not have a chance to participate in a research project in real work [18]. Pursuing research was challenging without prior research experience and confidence. Therefore, researchers advocated for interventions such as education, training, mentoring, academic-clinical collaborations, journal clubs, seminars, workshops, academic meetings, opportunities for experiential learning, and research facilitators to improve nurses’ capacity for research [18,20,21,22]. Research competence in nursing necessitated long-term investments in both research implementation and research capacity building [19].

One key element determining community nurses’ research abilities is the number of participants in research training during the last year. Community nursing administration may be to blame for this. Most community nurses in China are responsible for a large caseload. Community nurses were not required by the center’s chief nurse to publish any research or produce any manuscripts. Community nurses who work with university researchers on a study have the most interest in and motivation for research activation and research training. Face-to-face interviews with community nurses revealed that they had little opportunity to participate in research training because of their workloads and limited access to resources like time, money, and expertise. Clinical nurses may be more likely to participate in research if they have a significant patient-centered clinical question, strong peer support, and organizational backing [23]. The research ability among clinical nurses has become a role and requirement. It plays an important role in clinical nursing research. Consequently, policymakers should provide the required conditions to cultivate the research ability in nursing, such as motivation, infrastructure (e.g., material support, funding, research training), and collaboration [19,24]. It may be difficult for nurses to improve their research skills if they do not have adequate resources. Clinical nurses have challenges while attempting to undertake research due to a lack of funding, time, self-assurance, organizational backing, and/or recognition of the practical importance of the findings [18,25].

### 5.1 Nursing implications

The methodological design is a sequential qualitative–quantitative study, and it could better combine qualitative and quantitative data. This design could better help develop the instrument of research ability, with high reliability and validity. Most community nurses have only average or below-average research skills. Without financial backing for study, there is a lack of scholarly fervor among community nurses. As a result, it is critical to equip community nurses with resources to enhance their capacity for research. The Health Ministry should implement proper policies to enhance community nurses’ capacity for research. Community health center leaders should push their staff nurses to get involved in research by funding their education, publishing their work, and collaborating with academic researchers.

### 5.2 Limitation suggestions

The limitations of this study should be addressed. One limitation is due to the pandemic of COVID-19; this qualitative study was limited to only 15 community nurses, which is a significant constraint. The chief nurse at a community health service center is an ideal participant for a qualitative study’s first phase. Another limitation is that all participants selected are community nurses by using purposive sampling in phase 1 qualitative research; therefore, the results of this study are not representative. Further research should contain different participants (head nurse and general nurse) in different communities (public community and private community) in China.

## 6 Conclusion

A highly valid and reliable survey tool was created to evaluate the current situation and influencing factors of community nurses based on the narratives of participants, which was conducted by face-to-face interviews. Six main themes that appeared to express the cognition of the participants toward the research ability that the community nurses must master nowadays, included (1) literature review ability, (2) problem-solving ability, (3) research design ability, (4) research practice ability, (5) data analysis ability, and (6) manuscript writing ability. Most community nurses also had just rudimentary research skills. Among community nurses in Nanning, Guangxi, China, research ability was significantly influenced by age, level of education, years of work experience, number of research projects participated in, number of articles published in Chinese core journals or SCI, and number of participants in research training in the previous year. Therefore, this instrument may serve as a valid survey tool for gauging the current state and affecting variables of researchers’ abilities, and by gaining a deeper understanding of individuals’ research skills gaps, useful research training may be made available to students, nurses, and other workers. The dependability of the survey tool created in this study should be tested in a wider range of participants (nurses, nursing educators, nursing students, etc.). The goal is to have a better understanding of the existing state of affairs and the elements that influence the research abilities of various participants. The findings could inform the development of new strategies to improve the research skills of study participants.
